# Standardizing decellularization protocols for optimized uterine tissue bioengineering^☆^

**DOI:** 10.1016/j.reth.2024.12.011

**Published:** 2024-12-21

**Authors:** Edina Sehic, Lucía de Miguel-Gómez

**Affiliations:** a Department of Medical Cell Biology, Uppsala University, 75123 Uppsala, Sweden; b Laboratory for Transplantation and Regenerative Medicine, Sahlgrenska Academy, University of Gothenburg, Kvinnokliniken, Blå stråket 6, 40530 Gothenburg, Sweden; c Department of Obstetrics and Gynecology, Institute of Clinical Sciences, Sahlgrenska Academy, University of Gothenburg, SE-405 30, Sweden

**Keywords:** Decellularization, Bioengineering, Scaffold, Uterus

## Abstract

Bioengineering is applied in different areas, including women's infertility management. Among other approaches, decellularized tissues are being used to treat uterine disorders causing infertility. Biomaterials made from decellularized tissue consist of tissue-specific extracellular matrix and, as acellular scaffolds, are thought to be immune inert. Hence, they are good grafting candidates to replace and regenerate excised damaged uterine tissue to cure infertility. However, decellularization approaches differ among species and research groups, posing challenges for comparison and standardization. The diversity in data reporting and studied properties of the resulting decellularized scaffold make it even more difficult, especially when the ultimate goal is clinical translation. Thus, this review aims to critically assess whole uterus decellularization studies, extracting and comparing their main results and conclusions. After carefully evaluating the reviewed studies, we noticed that the vast majority base the uterus decellularization success and resulting scaffold efficacy on the DNA removal efficacy, while other crucial aspects, including the extracellular matrix integrity or immunogenicity, are underestimated. Thus, this review further proposes practical points for what should be considered and how results can be reported in studies involving whole uterus decellularization to facilitate comparison between studies and translational progress.

## Highlights

• Decellularized scaffolds need to maintain the extracellular matrix integrity.
• A balance between cell removal and extracellular matrix preservation is critical.
• The scaffold's biocompatibility and immunogenicity should always be considered.

## Graphical abstract

Decellularized tissues have emerged as a promising therapeutic approach to treat uterine disorders, as the resulting acellular extracellular matrix (ECM) is a good grafting candidate to replace and regenerate excised damaged uterine tissue, thereby addressing infertility. However, decellularization protocols differ among species and research groups (e.g., choice of decellularization agents), complicating efforts to compare and standardize procedures. There is also diversity in data reporting and studied properties of the resulting decellularized scaffolds. A thorough revision of the available literature suggests that beyond successful cell removal, the scaffolds' ECM integrity, biocompatibility, and immunogenicity should also be addressed.

**Figure:**
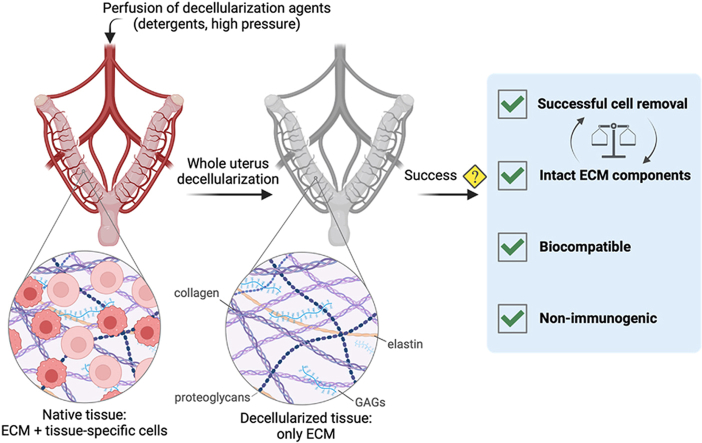

## 1 Introduction

Tissue engineering offers the potential to restore or replace damaged tissues and organs by combining different biomaterials, engineering approaches, biomolecules, and cell sources [1]. This approach is promising for addressing a wide range of medical conditions, including infertility and, more specifically, uterine disorders [2]. Uterine conditions such as adhesions, severe myomas, and scarring after surgical interventions can lead to absolute uterine factor infertility (AUFI), which is defined as a complete dysfunction or absence of the uterus, affecting up to 1:500 women of reproductive age [3]. Traditional treatments, including major surgeries, often fail to fully restore uterine function [4]. To date, uterus transplantation (UTx) has emerged as a viable cure for AUFI. However, it is limited by donor availability, risky live donor surgery, and immunosuppressive-treatment side-effects [5]. Therefore, uterine tissue engineering emerges as a promising alternative, aiming to create patient-specific, functional tissue substitutes that can replace or repair damaged uterine tissue, bypassing UTx challenges and providing a more compatible and accessible treatment. As a practical and significant step toward the goal of fully bioengineer a whole uterus, research focused on developing treatments for partial uterus repair using tissue-engineered materials, which could also treat less severe uterine conditions like non-severe intrauterine adhesions, myomas, or Caesarean section tissue recovery [4].

Both biological and synthetic materials have been explored, each presenting unique advantages and challenges [1]. Biological materials, such as decellularized tissue, preserve extracellular matrix (ECM) properties while cellular components, including genetic materials, from donor tissue are removed. The decellularized tissue and remaining ECM serve as a scaffold that can support cell attachment, growth, and differentiation, facilitating the regeneration into functional tissue [6]. Synthetic materials offer advantages such as precise control over structural and mechanical properties. However, they often fail to fully mimic the ECM composition found in the native tissue. In the context of uterine tissue engineering, both synthetic and biological approaches have been extensively explored. Materials like collagen-based rigid scaffolds [7], hyaluronic acid hydrogels [8], and decellularized tissue from various species [2] have been developed and transplanted into animal models of uterine damage. However, regarding the latter, there is no consensus on decellularization protocols or ECM evaluation methods, making cross-study comparison difficult. This issue also appears when decellularizing other organs, including cardiac [9] or intestinal submucosa [10] tissues. Thus, the lack of standardization among decellularization protocols for scaffold generation makes their further clinical application difficult to assess.

This review examines the most common whole-organ decellularization techniques used in uterine tissue engineering (also applicable to other tissues and organs), focusing on (i) DNA content evaluation, (ii) ECM-specific proteins, (iii) cytocompatibility and immunogenicity, and (iv) mechanical properties in the obtained decellularized scaffolds. Thus, contrary to previous reviews written in the field, we aim to propose that future uterus tissue engineering studies should use standardized parameters that enhance comparability, reliability, and efficiency of research and thereby accelerate technology transfer to clinical applications.

## 2 Decellularization challenges: the importance of analyzing ECM components

Decellularization aims to obtain an intact or minimally damaged ECM. However, the “decellularization paradox” [11], which highlights the challenge of balancing the removal of cellular components without compromising ECM integrity, is a crucial point in the field [12]. The ECM is vital for its role in providing cellular support. Its components, such as structural (collagen, elastin) and non-structural (fibronectin, laminin, tenascin) proteins, integrins, growth factors, and metalloproteinases, play crucial roles in cell behavior, influencing processes like cell adhesion, differentiation, and migration [13]. Therefore, comprehensive ECM analysis after decellularization is essential to ensure the scaffold's functionality in tissue engineering. Thus, the ideal ECM evaluation and analysis after decellularization should cover the remaining ECM composition and mechanical properties through quantitative and qualitative techniques. However, current literature often focuses primarily on remaining donor DNA concentrations, using only this parameter as a decisive checkpoint to determine decellularization success. While DNA removal is essential to reduce immunogenicity, it does not provide a complete picture of the remaining ECM status.

The diversity of existing uterus decellularization protocols and evaluation approaches further complicates the challenge for cross-study comparison. The reviewed articles herein (a total of 22) mainly use ionic (e.g., sodium dodecyl sulfate, SDS and sodium deoxycholate, SDC) [[14], [15], [16], [17], [18], [19]] and non-ionic detergents (e.g., Triton X100) and physical methods (e.g., high hydrostatic pressure) [20,21] as decellularizing agents. Sometimes, studies also combine [[22], [23], [24], [25], [26], [27]] or compare [[28], [29], [30], [31], [32], [33], [34], [35]] these different approaches (Table 1). Each method has distinct effects on the ECM components, necessitating careful protocol selection and optimization to achieve desired outcomes. Also, using detergents, especially ionic ones, always entails the risk of chemical residuals if the tissue is not adequately rinsed after decellularization [36,37]. Additionally, anatomical and morphological uterine differences across species need to be considered to ensure the ECM's applicability in various biological contexts [38]. The scientific community has also worked with other approaches, such as decellularizing only small uterine segments [39], the endometrium [40], or the myometrium [41]. However, this review focuses on studies utilizing whole uterus decellularization.

**Table 1:** **DNA concentration before and after different decellularization protocols: data from reviewed studies that provided quantitative data.** When data was available, values were shown as ng of DNA per mg of tissue (wet or dry) and/or as % of remaining DNA compared with the native tissue (if available). ∗: Data recalculated or explicitly converted to % of remaining DNA for this review from published values. F/T: freeze/thaw; HPP: high hydrostatic pressure; N/A: no data available; NP: non-proliferating endometrium; P: proliferating endometrium; SDC: sodium deoxycholate; SDS: sodium dodecyl sulfate; T-X100: Triton X100; UDL: under detection limits.

Species	Decellularization protocols (with absolute values and % of remaining DNA)			Native tissue values	Units	References
Rat	DMSO, T-X100, PBS 819 (32 %)	DMSO, T-X100, dH2O **33 (1 %)∗**	SDC **161 (6 %)∗**	2574 (100 %)	ng/mg (wet), %	[31]
	SDS 96 (10,97 %)∗	HPP **53 (6,06 %)∗**		875 (100 %)	ng/mg (wet), %	[35]
	SDS (increasing concentration gradient) *DNA removal evaluation only by H&E staining*			N/A	N/A	[17]
	DMSO, T-X100, PBS 18 %	DMSO, T-X100, dH2O **UDL**	SDC **UDL**	2574 (100 %)	ng/mg (wet), %	[34]
	SDS (increasing concentration gradient) *Validated protocol* [17] - *only H&E staining*			N/A	N/A	[18]
	SDS + increasing concentration gradient of T-X100 *DNA removal evaluation only by H&E staining*			N/A	N/A	[19]
	DMSO, T-X100, PBS 420 (16,5 %)	DMSO, T-X100, dH2O **13 (0,5 %)**	SDC **38 (1,4 %)**	2545 (100 %)	ng/mg (wet), %	[32]
	DMSO, T-X100, PBS	DMSO, T-X100, dH2O	SDC	N/A	N/A	[33]
	*Validated protocols* [31,34] - *only H&E staining*					
	250 MPa **4,04 %**	500 MPa 12,00 %	980 MPa **4,14 %**	N/A	%	[20]
	Biorreactor, HPP **10 %∗**	Shaking, HPP 33 %∗	Static, HPP 55 %∗	N/A	%	[21]
	SDS, T-X100, PBS 0,60 %			N/A	ng/mg (dry), %	[25]

Rabbit		T-X100, SDS **4,81 %**		N/A	ng/mg (wet), %	[22]
	T-X100, SDS (NP tissue) **10 (4.7 %)∗**	T-X100, SDS (P tissue) 15 (6.8 %)∗		220 (100 %)	ng/mg (wet), %	[24]

Cat	SDS, T-X100	SDC, T-X100 x2	SDC, T-X100 x2 + DNAse I	N/A	ng/mg (wet)	[27]
	*Significant DNA decrease only after the last protocol– data only shown on graphs*					

Pig	T-X100, SDS (fresh tissue) 139 (8,85 %)	T-X100, SDS (F/T) **40 (2,55 %)**		1571 (100 %)	ng/mg (wet), %	[23]
	SDS, T-X100, dH2O 2.3			N/A	ng/mg (dry)	[26]

Cow and sheep	SDS, T-X100, PBS **24,3 (13,5 %)**	DMSO, T-X100, PBS 54,3 (30,17 %)	SDS gradient 44,7 (24,83 %)	180 (100 %)	ng/mg (dry), %	[28]
	SDS 4,5 (1,12 %)	SDC **2,3 (0,58 %)**	SDC, T-X100 **3,7 (0,93 %)**	398 (100 %)	ng/mg (wet), %	[29]
	SDS	SDC	SDC, T-X100	N/A	ng/mg (wet)	[30]
	*Significant DNA decrease, no differences among methods – data only on graphs*					
	SDC *Data not shown due to previous validation of the method*			N/A	N/A	[14]
		SDC **36,75 (1,28 %)**		2866 (100 %)	ng/mg (wet), %	[15]

Human		SDS **40,11 (1,62 %)**		2463 (100 %)	ng/mg, %	[16]

Analyzing the uterine ECM after decellularization is crucial for advancing the field. A thorough understanding of how different decellularization protocols affect ECM integrity can guide the development of standardized methods, improving reproducibility and accelerating progress in the field. Future research should focus on comprehensive ECM analysis techniques in combination with donor DNA quantification. Integrating advanced imaging techniques, proteomics, and biomechanical testing can provide a more comprehensive understanding of the ECM's condition post-decellularization. Additionally, evaluating the immunological responses and other safety measures at an early stage will facilitate clinical translation. This approach will enable researchers to develop more effective protocols, ultimately accelerating the creation of functional uterine tissue replacements for clinical use.

## 3 Key indicator: DNA content post-decellularization

As previously mentioned, the analysis of the remaining donor DNA in decellularized tissue is still a key indicator for evaluating the efficiency of decellularization despite the source tissue or organ. Effective decellularization should ideally remove cellular material, including DNA, to reduce the risk of a negative immune response in host tissue after engraftment [6]. Typically, and in the reviewed studies herein, DNA content is assessed using commercial kits, often combined with spectroscopy and/or electrophoresis to quantify double-stranded (ds) DNA concentration [31]. Histological techniques such as hematoxylin and eosin (H&E) staining, along with immunofluorescence stains like 4′,6-diamidino-2-phenylindole (DAPI), are commonly employed to visualize remaining cell nuclei, thereby validating the success of the decellularization process. These methods are well-established and appear in most of the reviewed studies (Table 1, Table 2).

**Table 2:** **Evaluation characteristics of the acellular uterine ECM after decellularization.** The table includes a summary of the most relevant characteristics evaluated by the reviewed articles in their obtained decellularized uterine materials. Specifications, examples of the assessment methods (including if they provide quantitative or qualitative data), and the specific reviewed works performing them are included. BCA: bicinchoninic acid; CAM: chorioallantoic membrane; DAPI: 4’,6-diamidino-2-phenylindole; DRG: dorsal root ganglion; GAGs; glycosaminoglycans; IF: immunofluorescence; IHC: immunohistochemistry; LC/MS: liquid chromatography-mass spectrometry; MTT: tetrazolium salt 3-(4,5-dimethylthiazol-2-yl)-2,5-diphenyltetrazolium bromide) – cell viability assay; SEM: scanning electron microscopy; TEM: transmission electron microscopy; VVF: Verhoeff Van Gieson.

Characteristics		Assessment methods	Analysis	References
Cellular content	**dsDNA**	Commercial kits for DNA extraction Absorption spectrophotometry for DNA quantification	Quantitative	[15,16,[20], [21], [22], [23], [24], [25], [26], [27],[29], [30], [31],^35^]
	**Cells**	IF – DAPI staining	Qualitative	[15,20,23,24,27,29]
		Histology – Hematoxylin and eosin	Qualitative	[[15], [16], [17], [18], [19],[23], [24], [25], [26], [27],^29^,^31^]

Acellular ECM content	**Total protein**	Commercial dye-based colorimetric assays – e.g., BCA, Bradford assay	Quantitative	[15,[22], [23], [24], [25],[29], [30], [31]]
		Whole proteome – e.g., LC/MS	Omics	[26,31]
	**Collagen**	Commercial dye-based colorimetric assays	Quantitative	[15,16,[20], [21], [22],^26^,^29^,^30^,^35^]
		Histology – e.g., Masson's Trichrome staining	Semi-quantitative	[15,16,20,23,[25], [26], [27],^29^]
		IF/IHC – anti-collagen antibodies	Semi-quantitative	[[16], [17], [18], [19],^23^,^26^,^30^]
		SEM/TEM – fiber distribution, quantification and thickness	Quantitative	[15,30,35]
			Qualitative	[16,19,20,23,25,27,29,31]
	**Elastin**	Commercial dye-based colorimetric assays	Quantitative	[15,20,26,29,31,35]
		IF/IHC – anti-elastin antibodies	Semi-quantitative	[16,23,26,30]
		Histology – e.g., VVF or Orcein staining	Qualitative	[15,20,27,29,30]
	**Laminin**	IF/IHC – anti-laminin antibodies	Semi-quantitative	[[17], [18], [19],^26^,^30^]
	**Fibronectin**	IF/IHC – anti-fibronectin antibodies	Semi-quantitative	[18,19,30]
	**GAGs**	Commercial dye-based colorimetric assays	Quantitative	[15,21,26,29,31]
		Histology – e.g., alcian blue staining	Qualitative	[15,27,29]
	**Vasculature integrity**	Stain perfusion, resin model	Qualitative	[23,29]

AcellularECM properties	**Biological properties**	Cytocompatibility – coincubation with cells (MTT assay), blood or sensitive tissues	Quantitative	[15,25,27,30]
		Axon regeneration potential	Quantitative	[30]
		Immunogenicity – in vivo animal transplantation	Quantitative	[15,32,33]
	**Mechanical properties**	Material testing machines – maximum load, Young's modulus, ultimate tensile strength	Quantitative	[15,16,20,22,25,29,31,35]

A review by Badylak et al. in 2011 suggested three criteria for assessing decellularization success: (i) less than 50 ng dsDNA per mg of ECM dry weight, (ii) DNA remnants with less than 200 base pairs in length, and (iii) lack of visible cell nuclei after DAPI or H&E stainings [42]. While these benchmarks offer valuable guidelines, they do not account for the remaining ECM's condition or directly assess residual DNA's immunogenic potential. Moreover, these thresholds have not been empirically validated regarding their ability to prevent the immune response. Despite the availability of these criteria, there has yet to be a consensus on decellularization guidelines, leading to significant variation in how DNA and also protein concentrations are reported, making cross-study comparisons challenging.

This discrepancy in data presentation results in substantial challenges. Most studies reported DNA values in terms of dsDNA mass per tissue weight (e.g., ng/mg), but there is an inconsistency in whether uterine tissue weight is measured wet or dry (Table 1). This discrepancy is critical as moisture content can vary between tissues and is influenced by storing conditions, among other factors [43]. Additionally, some studies express the DNA concentration as a percentage of decreased or remaining concentration relative to native tissue rather than providing absolute values (Table 1). Without standardized normalization practices, it becomes challenging to interpret the relative efficacy of uterus decellularization protocols.

Establishing common normalization standards could mitigate discrepancies arising from variations in tissue size and initial DNA content. To address this, we have extracted DNA concentration data from all reviewed articles and presented them in Table 1, converting data where possible to facilitate comparison [21,24,31,35]. While some studies report reducing the initial DNA content to 2 % after decellularization [15,16,25,29,31,32,34], others accept up to 4 % [20,[22], [23], [24]], 6 % [35], 10 % [21], or up to 13 % [28] compared to the native tissue levels. The critical consideration is whether the remaining DNA, regardless of its percentage relative to the native uterine tissue, the fragment sizes, or the absolute concentrations, can trigger the host immune system.

## 4 Extracellular matrix composition after decellularization

The ECM consists of two main components: fibrous proteins and proteoglycans. Evaluating these components after uterus decellularization is essential, as the primary goal is to maintain an intact acellular ECM capable of supporting regeneration and function [6]. Additionally, ECM integrity also implies maintaining intact vasculature conduits [23,29]. However, evaluating the ECM components after uterus decellularization presents significant challenges, as the techniques, target molecules, and data expression approaches vary among studies, making comparison challenging (Table 2).

### 4.1 Collagen

Collagen, the most abundant protein in the ECM, is extensively evaluated in decellularization studies. It acquires even higher relevance in the uterine tissue. Besides being responsible for tissue integrity and tensile strength, collagen cross-links are fundamental during pregnancy and delivery [44]. Evaluation methods for collagen range from massive proteomic analyses, or imaging using scanning electronic microscopy (SEM), to chemical staining, immunohistochemistry, and immunofluorescence assays (Table 2). From a qualitative perspective, collagen fibers are generally preserved in uterine scaffolds and evenly distributed but with lower density after decellularization.

These observations are supported by the few studies that quantitatively measured reduced fibers after uterus decellularization [35]. In contrast, SEM studies reported no differences in collagen fiber thickness [15,30] or alterations in the triple helical structure [20,23]. Despite these findings, the studies reviewed in this article consistently show that at least 50 % of collagen content in the uterine tissue is preserved after decellularization. However, it is important to note that the ECM contains multiple types (chains) of collagen [45], with different reported functions, and that not all reviewed studies measure the same type. Other studies focus on the soluble and insoluble fractions rather than the collagen chain type [15,29].

### 4.2 Elastin

Elastin, another crucial fibrous protein, is key in maintaining uterine tissue elasticity [46]. This protein is also crucial during pregnancy, as elastin significantly increases size to accommodate uterine growth [47]. Comparative studies on different decellularization protocols reveal substantial differences in elastin content and preservation, indicating that the method chosen for uterus decellularization significantly affects elastin integrity [27]. For instance, ionic detergents such as SDS and physical methods like high hydrostatic pressure have varied impacts on elastin integrity [35]. Similar to collagen, the evaluation of elastin post-decellularization lacks consensus regarding methodology. While some studies report preserved elastin levels [20,35], others account for a decrease in the uterine ECM [29,31]. Establishing uniform criteria, including standardized quantitative and qualitative assessment methods, would allow for a more accurate and reliable comparison between studies, facilitating reproducible research outcomes.

### 4.3 Laminin and fibronectin

Only a few studies have reported the uterine ECM content of laminin and fibronectin, which are proteins involved in cell adhesion, migration, and differentiation [6]. In the uterus, both proteins are crucial for embryo attachment and invasion of the endometrium [48]. The preservation of these proteins is vital for the functional integrity of decellularized tissues, as they provide necessary biochemical cues that influence cell behavior. Qualitative assessments, such as immunohistochemical staining, indicate that these proteins are generally well-preserved after uterus decellularization [18,19,23].

Quantitative studies further support the preservation of laminin and fibronectin levels after decellularization. Two independent research groups [16,26] employed various enzyme-linked-immunosorbent assays (ELISA) and mass spectrometry to ensure the levels of these proteins in the uterine ECM. Their results indicate that the content of laminin and fibronectin remains stable and comparable to native uterine tissues, reinforcing that the selected decellularization protocols effectively preserve these critical components. However, it is essential to note that comprehensive analyses of laminin and fibronectin are limited in the current literature, highlighting a significant knowledge gap.

### 4.4 Glycosaminoglycans

Glycosaminoglycans (GAGs), the most abundant proteoglycans in the ECM, are important in maintaining tissue's structural integrity and biochemical functionality. GAGs contribute to the ECM's viscoelastic properties, hydration, and resistance to compressive forces, making them critical for proper tissue function and regeneration [49]. Thus, it is also key in embryo implantation and pregnancy processes [50]. Quantitative evaluation consistently reports a significant decrease in GAG levels after uterus decellularization [26,29,31]. This reduction in GAGs content is also evidenced through alcian blue staining. However, the effectiveness of alcian blue staining in decellularized tissues requires further discussion, as the absence of cellular components limits the counterstaining options, complicating the accurate measurement of blue intensity and, consequently, GAGs content. A concerted effort to standardize GAG evaluation and optimize decellularization protocols will significantly benefit the field. By ensuring the retention of these essential proteoglycans, researchers could create more effective and reliable uterine ECM scaffolds.

## 5 Immunological considerations in decellularized uterine tissue

### 5.1 Damage-associated molecular patterns

Initially, decellularized materials were believed to be immune-privileged due to the removal of cellular components. However, complete cell removal without damaging the ECM is almost impossible [11]. Recent research suggests that these cell remnants, together with chemical residues and fragmented ECM molecules that remain in the scaffold after decellularization, can act as immune-reactive damage-associated molecular patterns (DAMPs) [33]. DAMPs are endogenous molecules released from damaged or dying cells that trigger an immune response. Hence, DAMPs should always be considered as the immune system recognizes them as tissue damage or stress signals, initiating inflammatory responses [51]. Overly aggressive decellularization protocols may inadvertently increase the immunogenicity of the ECM by generating more DAMPs, which could compromise the scaffold's biocompatibility [52].

Research has shown that less aggressive protocols can reduce the immune response [52]. For instance, a study by Padma and collaborators found that using a mild yet efficient detergent protocol with Triton-X100 for uterus decellularization was immunologically advantageous compared to the more aggressive SDC treatment [32]. The more aggressive decellularization treatment generated more DAMPs that even activated the immune response following engraftment in a genetically identical (syngeneic) animal model [33]. This advantage was attributed to donor DNA levels below 1 % of the original content. Despite these findings, the assumption that low DNA concentration equates to immune-inert scaffolds has led to a lack of comprehensive studies on the immune response during protocol optimization. Furthermore, potential traces of chemicals when using chemical decellularization strategies can also trigger DAMPs. Evaluation of these traces by sensitive methods, such as liquid chromatography, is also advisable when optimizing decellularization methods [36]. Establishing evaluations of immunogenicity as a standard practice in decellularization protocol development could significantly improve outcomes in tissue engineering and prevent adverse events in future translational studies.

### 5.2 Cytocompatibility and immunogenicity

This step is essential before clinical application. The scaffolds must be biocompatible and not induce significant immune reactions. Still, only a few reviewed studies address this issue and, again, the techniques used are diverse.

Cytocompatibility is often assessed through in vitro cytotoxicity assays, such as the colorimetric tetrazolium salt-based MTT assay, which evaluates cellular metabolic activity and is recognized in ISO 10993-5:2018 for the evaluation of medical devices [53]. Despite the pre-clinical nature of all reviewed articles, barely two studies used this technique, demonstrating that the scaffold did not inhibit stem cell growth from either rabbit [25] or human [29] origin. Other studies have evaluated the scaffold's cytocompatibility by assessing its interaction with sensitive tissues like chicken embryos [15,30] or sperm [27], showing no adverse effects on development and viability. Evaluating hemocompatibility also provides insights into scaffolds' biocompatibility [53]. Although ISO 10993-4:2018 only requires hemocompatibility tests for materials in direct contact with blood (e.g., arterial scaffolds), conducting these tests before clinical translation would be advisable. A recent study confirmed the non-hemolytic properties of rat-decellularized uterine scaffolds, highlighting the importance of this evaluation before clinical translation [25]. Yet, examples of alternative parameters to be analyzed are thrombogenicity or clotting time [54] and complement activation system [55].

Immunogenicity is typically evaluated in vivo using animal models or in vitro with immune cells. In uterine research, in vivo testing has primarily involved quantification of infiltrating immune cells post-transplantation [15,32], with a focus on infiltrating macrophage and T-cell subtypes. Still, in vitro studies, such as using peripheral blood mononuclear cells [56], are preferred to reduce animal use.

## 6 Mechanical properties of decellularized scaffolds

The ECM is widely recognized for its function as a supportive framework, providing mechanical stability to tissues and organs. The mechanical properties of the ECM are crucial not only for maintaining structural integrity but also for influencing cellular behaviors such as stem cell differentiation [57]. In uterine tissue engineering, evaluation of the mechanical properties of decellularized materials is particularly important, as they must be capable of supporting a pregnancy [58]. Despite this relevance, most reviewed studies do not evaluate these parameters, and there are discrepancies regarding which specific parameters should be measured among those that do.

The Young's (or elastic) modulus is the most evaluated parameter that measures the scaffold's stiffness [59]. High hydrostatic pressure reduces Young's modulus [20,35], while detergent-based protocols often increase it [16,35]. Other important properties include tensile strength (the maximum force a scaffold can withstand before breaking), elasticity, and rupture strength (the point at which the scaffold will break). Besides, the maximum working load, that reflects the scaffold's capacity to withstand physiological forces, is often reduced after decellularization [15,29,31]. However, making comparisons among studies is challenging due to variations in measurement techniques and experimental conditions.

The dynamic and cyclic nature of the uterus, influenced by fluctuating estradiol levels, adds another layer of complexity to the evaluation of mechanical properties (and all the other reviewed variables). A recent study demonstrated that the Young's modulus of decellularized bovine endometrial tissue decreased after decellularization, but only in the luteal phase, resulting in a stiffer scaffold than those from the follicular phase [60]. The authors also reported a reduction in both the storage modulus (E’; how much energy is required to deform the scaffold) and the loss modulus (E’‘; indicating the proportion of rigidity due to viscous flow) during the follicular phase [59,60]. However, this study was limited by a small sample size (n = 3 per experimental group), highlighting the need for further research to validate these findings.

Evaluating mechanical properties is crucial for developing functional uterine scaffolds. Future research should focus on comprehensive mechanical assessments that consider physiologically dynamic conditions.

## 7 Optimal analysis of a decellularized uterine scaffold: final conclusions

Based on the information discussed, we suggest a list of points to consider that includes the minimal analyses we believe DC uterine scaffolds should undergo (Table 3). We have classified these parameters as essential (E) or desirable (D) information for, under our understanding, a proper characterization of these scaffolds (classification system adapted from Ref. [61]). Certainly, exhaustive compliance with all suggested items is not necessary, and it needs to be put in context.

**Table 3:** **Points to consider after decellularizing uterine tissue: a proposal towards standardization.** Based on the reviewed articles and available literature, the list includes the minimal information we consider should be addressed before considering a DC scaffold as a suitable biomaterial. The parameters to evaluate, the required results, and the grade of importance of the proposed techniques are covered; of course, they should always be put into the research group's context. D: desirable information; DC: decellularized; ds: double-stranded; E: essential information; GAGs: glycosaminoglycans; H&E: hematoxylin and eosin; IR: immune response.

Item to check		Evaluation		Required results	Importance
1	**Cellular content**	dsDNA concentration (relating to dry tissue weight; absolute values and % regarding the native tissue)		Max. 10 % in DC vs. native tissue	E
		Nuclei imaging (H&E and/or DAPI)		Absence of cell nuclei	D

2	**ECM content**	Collagen	Concentration	Min. 50 % in DC vs. native tissue	D
			Advanced imaging	Normal thickness and distribution of the fibers	**E**
		Elastin, fibronectin and/or laminin	Concentration	Min. 30 % in DC vs. native tissue	D
			(Advanced) imaging	Normal distribution of the fibers	**E**
		GAGs	Concentration	Min. 40 % in DC vs. native tissue	D
			(Advanced) imaging	Normal distribution	**E**
		Total protein content - proteomics		Preservation of key proteins	D
		Evaluation of vasculature integrity		Intact vessels	D

3	**ECM biological properties**	Cytocompatibility		Ensure cell/tissue viability after incubation with DC tissue	**E**
		Bioactivity evaluation (e.g., angiogenesis or neurogenesis assays)		Significant increase the specific activity in comparison with a non-DC biomaterial	D
		Immunogenicity		Inert behaviour (not or attenuated IR trigger)	**E**

4	**ECM mechanical properties**	Stiffness – Young's modulus		Maintained in DC vs. native tissue	**E**
		Elasticity – maximum working load			D
		Other properties (compressive strength, elongation at fracture)			D

First, the specific characteristics of the starting or native uterine tissue need to be considered, as uterus anatomy and morphology vary among species. The estrous phase should also be addressed. Most reviewed studies did not include this parameter in their methods section. One of the few examples is Campo and collaborators, who decellularized proliferating and non-proliferating uteri and reported differences in the DC scaffold characterization [24]. Second, the authors have to consider the decellularization paradox [11]. It would be advisable to evaluate if, e.g., compromising DNA content while improving mechanical properties or collagen preservation is worth it as long as immunogenicity remains the same. Regarding the latter, immunogenicity assays become indispensable before clinical translation. Thus, efforts should be directed toward practical detection methodologies for chemical residues, more efficient rinsing techniques after decellularization and sterilization, or even more immunocompatible scaffold sources, as previously suggested by Kasravi and colleagues [52]. Further, standardized methods to assess batch variability, scaffold safety, and functional performance will be necessary for clinical approval, as these will ensure reproducibility, reduce risk, and provide consistent therapeutic outcomes. Establishing common standards for tolerable immunogenicity and scaffold performance will facilitate regulatory alignment and enhance the likelihood of these materials researching clinical use. Finally, there is a need for standardized data reporting. Authors are encouraged to use international units and provide raw data to apply further calculations or conversion factors.

The herein review focuses on the obtention of decellularized uterine scaffolds and how to characterize them properly. Still, the further use of these materials often involves repopulation with specific types of cells. One common approach is using recipient-derived stem cells or commercially available cell sources [62]. Thus, the scaffold's cytocompatibility and recellularization possibilities should be carefully evaluated [53]. Other more specific aspects such as cell source and quantity, repopulation method (e.g., injection, perfusion), conditions (pressure, temperature), or incubation times need to be carefully addressed [62].

In conclusion, this review emphasizes the need for comprehensive and standardized evaluation methods in uterine tissue engineering, particularly focusing on the decellularization process. Ensuring minimal DNA content, while preserving the ECM proteins, and maintaining mechanical properties are critical for creating functional tissue scaffolds. Researchers should provide comprehensive details regarding the methods used for DNA and protein concentration analysis, including units of measurement, normalization procedures, and any relevant conversion factors applied. Transparent reporting practices enable readers to interpret comparative analyses accurately. By establishing clear evaluation criteria, the field can advance toward developing more effective and reproducible decellularization protocols, ultimately accelerating the translation of these technologies into clinical applications for treating uterine disorders and infertility.

## Funding

The authors received funding from the 10.13039/501100014552Adlerbertska Research Foundation and the Hjalmar Svensson Research Found.

## Declaration of competing interest

The authors declare that they have no known competing financial interests or personal relationships that could have appeared to influence the work reported in this paper.
